# Introduction of digital reporting platform to integrate community-level data into health information systems is feasible and acceptable among various community health stakeholders: A mixed-methods pilot study in Mopti, Mali

**DOI:** 10.7189/jogh.11.07003

**Published:** 2021-03-30

**Authors:** Karen Kirk, Tracy L McClair, Sina Pascal Dakouo, Timothy Abuya, Pooja Sripad

**Affiliations:** 1 Population Council, New York, New York, USA; 2 Population Council, Washington, D.C., USA; 3 Aga Khan Foundation, Bamako, Mali; 4 Population Council, Nairobi, Kenya

## Abstract

**Background:**

Integration of community-level health data within Mali’s web-based District Health Information System (DHIS2) is underexplored. This study conducted in Mopti, Mali examined challenges and enablers affecting integration and investigated how digital technology optimizes data quality, availability, and use.

**Methods:**

This pre-post mixed-methods study compared community health workers’ (CHWs’) experiences reporting routine community-level data using the DHIS2 digital application on tablets and paper forms. 141 CHWs participated in quantitative surveys and focus group discussions at baseline and endline. In-depth interviews were conducted with 18 and eight CHW supervisors and 12 and 11 other stakeholders at baseline and endline, respectively. We calculated changes in CHW performance, and job satisfaction among intervention and comparison groups using the difference-in-difference (DID) estimator controlling for baseline characteristics. Routine longitudinal DHIS2 data described timeliness and completeness of CHW reporting. Thematic analysis of qualitative data explained implementation challenges and enablers, and considerations for data use.

**Results:**

The median number of health talks and household visits among intervention group CHWs increased from baseline to endline compared to the comparison group (DID estimator; *P* < 0.05 for both), as did aspects of job satisfaction (satisfaction with opportunities to contribute ideas to improve services and coordination with supervisors and stakeholders, *P* < 0.1). CHWs reported high levels of comfort and confidence navigating the tablet for data collection and on-time reporting. While CHWs experienced challenges –tablet quality, limited network connection and increased workload, they preferred the digital to paper system. Stakeholder, supervisor and CHW roles in data review and decision-making appear unchanged from baseline to endline, though some supervisors found the tablets improved data timeliness and completeness. Routine longitudinal DHIS2 data confirm high rates of data timeliness and completeness before and after the intervention, with little or no change over time.

**Conclusions:**

CHW tablet use for data collection and reporting is feasible and desirable, however, program and policy changes are needed for this to be a fully-functional system. Future efforts need to consider how to ensure site-level network connectivity; quality, compatibility and functionality of digital technology; and routine supportive systems for CHWs and community health actors on data use.

Community health data are increasingly required in low- and middle-income countries (LMICs) seeking to monitor service delivery, measure performance, improve accountability and make health programming decisions [1-3]. However, health information systems (HIS) are challenged by inadequate staffing, uncoordinated reporting requirements, and lack of data ownership [4]. In many sub-Saharan African countries, health systems continue to use paper-based systems for health data, despite evidence showing that this contributes to poor data quality and compromises health service delivery [5-7].

Initiatives to reform paper-based HIS through digitalization have observed cost reductions and more timely delivery of health care services as well as managers and decision-makers being able to use disaggregated data in optimizing health services [8,9]. However, inadequate financing, weak infrastructure, limited human resource capacity, fragmented organizational structures, and diverse stakeholder interests have posed challenges to digitalization of HIS [10,11]. While digital health interventions are recognized as promising strategies to strengthen health systems, unanswered questions remain [12-14]. A consultative review and the “WHO guideline on health policy and system support to optimize community health worker programmes” identify the use of digital tools to strengthen community health data and programming as a priority area [12,15].

Mali, like many countries, has adopted a free, web-based, open-source HIS, the District Health Information System (DHIS2), for aggregation and analysis of health service data; however, community health workers (CHWs) continue to collect and report data using a paper-based system due to logistical and human resource challenges. Over 50 countries have deployed the DHIS2 in health facilities, but there is limited experience with its integration at the community level [16,17].

Within the USAID-funded Integrating Community Health Collaboration, the Aga Khan Foundation (AKF) implemented the Soins Essentiels dans la Communauté Project (SECPro) to improve community health and reduce maternal, newborn, and child mortality by strengthening the implementation of Mali’s national basic community health care strategy (SEC). As part of SECPro, AKF, in collaboration with the Population Council’s Frontline Health project, sought to examine the challenges and enablers that affect integrating community-level health data into DHIS2 and explore how technology could optimize the quality, availability, and utility of that data. Underlying this study is a focus on data use as a critical domain within the CHW Performance Measurement Framework [18].

The objectives of the study were to assess the effect and feasibility of implementing a digital data collection system at community level, specifically baseline-endline differences in (1) CHW ability and willingness to carry out their roles and responsibilities (2) timeliness and completeness of data reporting using the digital system, and (3) experiences using data from a digital community-based information system.

## METHODS

### Study design

This study uses a pre-post mixed-methods design that compared CHWs’ experiences using the DHIS2 digital application with that of paper forms to collect and report routine community health data. The study conducted quantitative interviews with CHWs to gauge changes in performance and experiences with community health data collection and reporting. Routine HIS data were also analyzed for trends in timeliness and completeness. Qualitative data were used to explore and gain a deeper understanding of the intricacies of CHWs’ experience collecting and reporting health data on tablets, describe data use for decision making and corroborate and explain quantitative findings.

### Intervention and study sites

Responding to inefficiencies in CHW data collection and reporting, AKF proposed to pilot test the use of the DHIS2 tablet-based application, which would improve the timeliness and completeness of community-level data submission. The existing paper data collection and reporting process requires completing individual forms for each client and marking register books, depending on services sought. These paper forms are supplied by the community health association (“Association de Santé Communautaire”, ASACO). At the end of each month, the CHW compiles all the client information into an aggregate report that is submitted in person to their supervisor, the directeur technique du centre (DTC) at the community health center (centre de santé communautaire, CSCOM) who reviews for quality, gives feedback, and enters aggregated monthly data from all CHWs they supervise into the DHIS2 platform.

CHWs in the intervention sites, Mopti and Djenné districts, received tablets with the DHIS2 application. However, due to concerns from the Ministry of Health (MOH) regarding the potential for lost data, the CHWs continued collecting and reporting data using the existing paper-based system in addition to entering the data into the tablet. AKF conducted the initial training and follow-up visits with CHWs and DTCs on tablet use for data collection and reporting. During these trainings, the AKF team members were accompanied by local representatives of the regional MOH and community.

The comparison sites, Bandiagara and Bankass districts, continued reporting data using the paper-based system only.

Study sites were selected based on where AKF was implementing the SECPro project and in consultation with local stakeholders. Comparison sites were selected with similar geographic, cultural and health characteristics as the intervention sites.

### Study participants

In the four study districts, we implemented quantitative surveys and focus group discussions (FGDs) with CHWs, and in-depth interviews (IDIs) with DTCs and other stakeholders at baseline and endline (**Table 1**). For the quantitative survey – with the exception of Bankass – all CHWs were recruited through gatekeepers including NGO and government liaisons. All recruited CHWs consented to participate in the study. We excluded health areas in Bankass district that were already enrolled in another study focusing on CHWs and integrated community case management [19]. All surveyed CHWs were invited to participate in FGDs. In each district, DTCs and stakeholders who are members of the district management team (Medical Chiefs, HIS officers, SEC Focal Points), community-level health representatives (ASACO and members of the Local Federation of Community Health Associations, FELASCOM) and the administration (Prefect) were invited to participate in IDIs. These stakeholders were interviewed if they were available at the time of the data collection in their district or community. Our primary outcomes include health data completeness and timeliness at district level and CHW ability to routinely and accurately submit community health data reports in the intervention and comparison arms.

**Table 1:** Data collection summary

	Baseline		Endline	
	**Intervention**	**Comparison**	**Intervention**	**Comparison**
Community health worker surveys*	62	79	62	79
Community health worker focus group discussions	4	5	4	4
Supervisors (Directeur Technique du Centre) in-depth interviews	9	9	4	4
Stakeholder in-depth interviews	4	8	5	6

*Baseline and endline respondents were matched, those who did not have both baseline and endline data were excluded (n = 11).

### Data collection

Data collectors were identified and recruited through a job notice published online in Mali, describing education requirements (Baccalaureate or above in a related field), professional experience (previous data collection experience, familiarity with mobile data collection, etc.) and proficiency in French and at least one local language.

Selected data collectors were trained on the study purpose, ethical considerations for research, and use of the study tools. Data collection was conducted over five days in each of the selected health districts. At recruitment, data collectors explained the scope and purpose of the study and received informed consent from all participants. Interviews were conducted primarily in French or Bambara. Some data collectors were proficient in other local languages and during a few FGDs, the respondents provided clarification in other languages when French and Bambara were not sufficient. Two data collectors were present for all qualitative interviews; one facilitated the interview while the other took notes to assist the transcription. The qualitative interviews were conducted in private or semi-private settings convenient for participants and sessions were audio-recorded, transcribed, and translated. Quantitative surveys were administered via a digital questionnaire installed on ARCHOS tablets using the Ona.io platform. Baseline and endline data were collected September-October 2018 and January 2020, respectively.

### Quantitative analysis

#### Measures

Sociodemographic characteristics include age (continuous), education (primary, secondary, vocational/professional, tertiary/superior), currently living in village where working as a CHW (yes vs no), and experience with smartphone/tablet (a lot, some, a little, none).

CHW performance was measured by self-reported number of health talks and number of household visits in the last month. Additionally, CHWs were asked about the number of hours they typically worked in a week. With regards to reporting, CHWs were asked to self-report if they transmitted data on time last month.

Six items were used to assess job satisfaction as it relates to data use and decision-making. Response options were: very unsatisfied, unsatisfied, satisfied, and very satisfied. We reported respondents’ level of satisfaction with the following: workload in relation to time available; opportunities to contribute ideas to improve services; coordination with DTC, ASACO, other members of CSCOM; participation in planning of services in catchment area; processes by which decisions are taken up by district officials; and timeliness with which decisions are implemented.

At endline, participants assessed their experiences reporting data via DHIS2, answering questions about technology training, confidence in using technology and the DHIS2, changes in workload, preferred method of reporting, and difficulties with mobile health data collection.

#### Statistical analysis

All statistical analyses were conducted in Stata v15 (StataCorp LLC, College Station TX, USA). Prior to conducting statistical analyses, we matched participant IDs at baseline and endline to ensure accuracy of paired data. Two participants at baseline and nine participants at endline were dropped because they did not have data for both, likely because they were not employed as CHWs in the community at one of the two points in time.

We examined frequencies of baseline characteristics among intervention and comparison groups, and compared differences using *t* tests for continuous variables and χ^2^ tests and for binary and categorical variables, as well as Fisher’s exact tests for when cell counts had less than five observations. We calculated changes in CHW performance, reporting, and job satisfaction from baseline to endline among intervention and comparison groups. We tested for significance in these changes using McNemar’s test for binary variables (exact test for cell counts less than five), Wilcoxon signed rank tests and paired *t*-tests for continuous variables. We calculated the difference-in-difference (DID) estimator controlling for baseline characteristics that were significantly different between groups. When the parallel trends assumption was not met, we used the Abadie semi-parametric DID estimator [20]. For the DID in job satisfaction between the two groups, a *P-*value of <0.1 was considered significant due to these indicators being more nuanced. Finally, we calculated frequencies of experience of DHIS2 among intervention participants at endline only.

Routine longitudinal DHIS2 data were used to describe timeliness and completeness of CHW reporting in pre-intervention, intervention, and post-intervention periods.

### Qualitative analysis

Digitally-recorded IDIs and FDGs were transcribed, translated, and checked for meaning by native French and Bambara speakers. Study researchers inductively and deductively developed a codebook based on the themes in the data and were explicitly asked about in the interview guides. Codebook themes were discussed and agreed upon within the study team. One member of the team then applied the codes and wrote accompanying memos to contextualize quotations in NVivo 12 (QSR International (Americas), Burlington, MA, USA). Thematic analysis of the data additionally sought to explain quantitative findings. Data quality control and logistical and technical management of the teams were led by the Monitoring & Evaluation Officer from SECPro at AKF, Mali.

### Ethics

Ethical approval for this study was received from the Population Council’s Institutional Review Board in New York, USA (p864) and the ethical committee of the National Public Health Research Institute in Bamako, Mali (n02013-1223/MS-SG) (le comité d’éthique de l’Institut National de Recherche en Santé Publique (INRSP)).

## RESULTS

### Community health worker characteristics

The baseline CHW characteristics by intervention and comparison sites are summarized in **Table 2****.** Overall, mean age in the intervention group was significantly higher than in the comparison group (32.2 vs 29.6 years, respectively). Most CHWs were male; more than half in the intervention group and more than two-thirds in the comparison group. Mean number of years working as a CHW was 4.7 and did not differ between intervention and comparison groups. Education status differed significantly between intervention and comparison groups. Notably, twice as many CHWs in the comparison group went to vocational/professional school compared to the intervention group. Finally, self-reported experience with smartphones and tablets varied significantly by site. Approximately half in each site reported “a lot” of experience with smartphones and tablets, however, 15% of those in the comparison group had no smartphone or tablet experience compared to only 1% in the intervention group.

**Table 2:** Baseline characteristics of community health workers (CHW)

	Intervention (n = 62)	Comparison (n = 79)	Total (n = 141)	*P*-value
	n (%)	n (%)	n (%)	
**Mean age (standard deviation)**	32.2 (9.3)	29.6 (5.6)	30.8 (7.5)	0.006
**Gender (female)**	30 (48.4)	24 (30.4)	54 (38.3)	0.029
**Education level:**				
Primary	33 (53.2)	26 (29.6)	59 (41.8)	0.006
Secondary	16 (25.8)	22 (25.0)	38 (27.0)	
Vocational/professional	12 (19.4)	38 (43.2)	50 (35.5)	
Tertiary/superior	1 (1.6)	2 (2.3)	3 (2.1)	
**Mean number of years working as a CHW (standard deviation)**	4.6 (1.9)	4.7 (2.4)	4.7 (2.2)	0.795
**Currently live in village where working as community health worker**	59 (95.2)	62 (78.5)	121 (85.8)	0.004
**Experience with smartphone/tablet:**				<0.001
A lot	32 (52.5)	40 (51.3)	72 (51.1)	
Some	11 (18.0)	0 (0.0)	11 (7.8)	
A little	17 (27.9)	23 (29.5)	40 (28.4)	
None	1 (1.6)	15 (19.2)	16 (11.3)	

### CHW ability and willingness to fulfill their role

Comparing baseline and endline results for CHWs in both sites, we identified some areas related to CHW performance, reporting, and job satisfaction that exhibit significant differences (**Table 3**).

**Table 3:** Changes in community health worker performance, reporting, and job satisfaction

	Intervention (n = 62)			Comparison (n = 79)			Difference-in-difference estimator*	
	**Baseline**	**Endline**	***P*-value**	**Baseline**	**Endline**	***P*-value**	**Estimator**	***P*-value**
Number of health talks held in last month, median (Interquartile Range)	3 (2-5)	5 (4-6)	<0.001	4 (2-5)	2 (1-4)	0.001	3.867	0.014
Number of household visits made in last month, median (interquartile range)	4 (3-10)	18.5 (15-27)	<0.001	3 (2-6)	5 (2-9)	0.336	12.043	<0.001
Mean number of hours working in a typical week (standard deviation)	41.9	58.0	<0.001	52.1 (12.1)	54.3 (8.6)	0.123	11.361	<0.001
**% of community health worker satisfied with…**	**n (%)**	**n (%)**		**n (%)**	**n (%)**			
Workload in relation to time available	48 (77.4)	55 (88.7)	0.144	58 (73.4)	69 (87.3)	0.016	-0.039	0.693
Opportunities to contribute ideas to improve services	50 (80.7)	58 (93.4)	0.022	71 (89.9)	71 (89.9)	1.000	0.101	0.084
Coordination with supervisors, Community Health Association (ASACO), other members of community health center (CSCom)	50 (80.7)	59 (95.2)	0.023	71 (89.9)	63 (79.8)	0.077	0.162	0.064
Participation in planning of services in catchment area	53 (85.5)	56 (90.3)	0.607	73 (92.4)	68 (86.1)	0.227	0.087	0.219
Processes by which decisions are taken up by district officials	48 (77.4)	45 (72.6)	0.678	63 (79.8)	55 (69.6)	0.201	-0.140	0.281
Timeliness with which decisions made at meetings are implemented	40 (64.5)	43 (69.4)	0.549	55 (69.6)	54 (68.4)	1.000	-0.109	0.433

*Multivariate analyses of difference-in-difference estimator (interaction between time point and study group) controlling for age, gender, education, living in village where working as a CHW, and smartphone/tablet experience.

#### CHW performance

The relationship between the introduction of tablets for CHW data collection and changes in CHW performance (frequency of health talks and household visits) is unclear. The quantitative data show modest improvements in some CHW performance indicators among CHWs in the intervention sites (**Table 3**). The median number of health talks in the month prior to data collection from baseline to endline increased in the intervention group (3 to 5 visits respectively, *P* < 0.001) and decreased in the comparison group (4 to 2, *P* < 0.01), with a significant DID estimator of 3.867 (*P* = 0.014). The median number of household visits conducted in the last month significantly increased from baseline to endline in the intervention group (4 to 18.5, *P* < 0.001), with a significant DID estimator of 12.043 (*P* < 0.001).

As expected, one area of CHW performance that changed for the intervention group was the mean number of hours working in a typical week which increased significantly from baseline to endline in the intervention group (41.9 to 58 hours, *P* < 0.001). Since the CHWs in the intervention sites were asked to enter data into the DHIS2 App in addition to the routine paper data collection and reporting system, this increase in workload and working hours was expected. In the comparison group, there was no change in the mean number of hours worked in a typical week (52.1 hours at baseline vs 54.3 hours at endline). The DID between comparison and intervention groups was significant (DID estimator = 11.361, *P* < 0.001). The increase in workload was expressed by a CHW:

“[Using the digital system] has increased my workload because I have a big village. For example, you have 60 children per month, you register each child on an individual card, in the register and in the tablet, then you conduct the visit […] I've cut off part of it, what I've got on the card, I'm not going to see it on my tablet, if I have to put it all on the tablet, I'm going to have sleepless nights.” *– CHW, Endline FGD, Intervention*

Even though CHWs in the intervention sites indicated that they were doing more work, one FGD respondent described the tablet as a status symbol that came with respect from village leaders. This positive reaction from prominent community members in response to the CHWs using tablets could potentially influence CHWs willingness or interest in conducting home visits or health talks in the community.

“When we were given the tablets and I went with them to my site, I showed the village chief and the imam, they said, ‘our doctor *[indicating CHW]* has become a chief now’, they were very pleased.” *– CHW, Endline FGD, Intervention*

#### CHW job satisfaction

CHWs’ reported job satisfaction marginally increased from baseline to endline in two domains in the intervention group (**Table 3**). Significantly more CHWs said they were satisfied with opportunities to contribute ideas to improve services (80.7% vs 93.4%, *P* < 0.05) and coordination with DTC, ASACO, and other members of CSCOM (80.7% vs 95.2%, *P* < 0.05). The DID in opportunities to contribute ideas to improve services and satisfaction with coordination among these CSCOM stakeholders were significant at the *P* < 0.1 level (DID estimator = 0.101, *P* = 0.084; DID estimator = 0.162 *P* = 0.064, respectively).

Despite reported positive changes in CHW job satisfaction, these themes did not readily emerge in the FGDs with CHWs from the intervention sites. Overall, the qualitative interviews describe frustrations with the ASACO although some CHWs describe their ability to make some temporary decisions for their communities including decisions related to selection of topics for health talk.

“Since we have been CHWs, the forms have been in short supply. The ASACO does not give us any forms. Now they have come with a new report […] (and) it is up to you to make your own copies, they give you one and that's it, at the end of the month it's up to you to make your copies.” *– CHW, Endline FGD, Intervention*

“In our community, we can make certain decisions, but they won't be long term. Whatever we decide will stay in our site and may not be taken to the next level sometimes.” *– CHW, Endline FGD, Intervention*

In the comparison sites, CHWs more explicitly expressed their collective lack of satisfaction in their work.

“CHW 4: We're in the job, and we like our job. But to say that we are happy or satisfied with this work: never.

CHW 1: We are not satisfied with this job. If there was satisfaction, a lot of people wouldn't quit. And among us, many people would want to quit if there were other opportunities.” *– CHW, Endline FGD, Comparison*

### Timeliness and completeness of health reports in DHIS2

Effects of tablet introduction on data quality and completeness were mixed from the DTC perspective. Although some acknowledged improvements, others found that CHWs “don’t enter everything they are doing in the tablet.”

“[The tablet] improved the (data) completeness; it's not 100% anyway, it improved a little bit the completeness compared to the beginning, the quality also […]. When you compare at the very beginning and now, it's not the same thing.” *– DTC, Endline IDI, Intervention*

**Figure 1:**
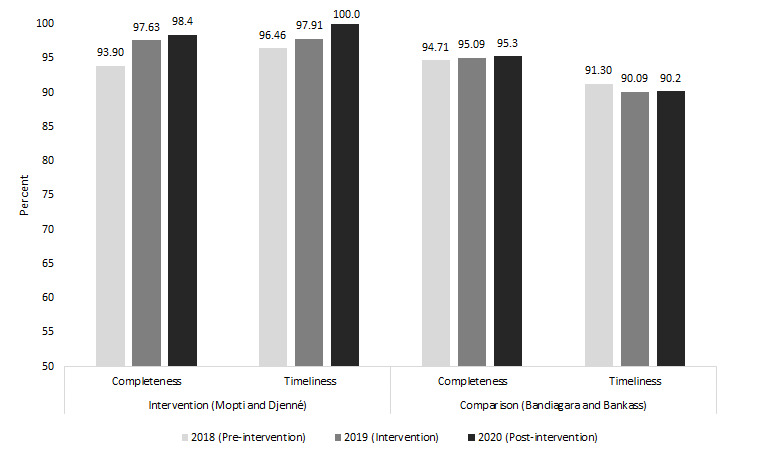
Average rate of timeliness and completeness of monthly community health worker reports from DHIS2 by year.

The quantitative survey results showed that CHWs reported high levels of comfort and confidence navigating the tablet for data collection and reporting and 100% stated that they submitted their reports on time last month (**Table 4**). Routine longitudinal DHIS2 data confirm overall high rates of data timeliness and completeness before and after the intervention, with little or no change over time (**Figure 1**). The increase in on-time and complete rates observed in the intervention sites has been sustained beyond the project.

**Table 4:** Experience with DHIS2 among intervention participants at endline (n = 62)

% of community health worker reporting	n (%)
Having been trained on DHIS2 app	62 (100.0)
Currently using tablet/smartphone to submit reports	62 (100.0)
Having been supervised about data collection using DHIS2 app	62 (100.0)
Transmitting data on time last month	62 (100.0)
Finding it easy to report data using tablet	56 (90.3)
Comfortable/confident in ability to use DHIS2 tracker app to report health data from their community	55 (88.7)
Comfortable/confident in ability to use tablet to record new client health data	54 (87.1)
Able to access and review data on tablet after it’s submitted	52 (83.9)
Comfortable/confident in ability to use tablet to access existing health records	49 (79.0)
Entered all of last month’s data in tablet	29 (46.8)
**Preferred method for data reporting:**	
Tablet/smartphone	57 (91.9)
Both (tablet and paper)	5 (8.1)
Paper tools	0 (0.0)
Change in workload since starting to use tablet to collect data:*	
Increased	42 (67.7)
Decreased	20 (32.3)
**Experience difficulties in reporting mobile health data:**	
Poor network connectivity	54 (87.1)
No phone/data credit	51 (82.3)
Battery not charged	43 (69.4)
Tablet damaged or lost	4 (6.5)

DHIS2 – District Health Information System

*No participants responded that their workload stayed the same.

### CHW and systems perceptions and experiences of DHIS2 integration of community health data

**Table 4** presents CHWs experiences in relation to the introduction of tablets for CHWs’ data collection and DHIS2 data reporting. In-depth interviews with DTCs and other stakeholders in the local health system show a more robust depiction of the overall changes felt in the intervention sites.

#### CHW experiences using tablet to report data

All intervention CHWs in our sample, trained and supervised on the DHIS2 app, were currently using a tablet or smartphone to submit reports on time with the majority (79%-90%) expressing confidence in using the tablet, including for recording new clients, reporting health data as well as being able to access and review data on their tablet after it’s submitted (**Table 4**). However, CHWs reported qualitatively that the training for data collection was inadequate.

“We learned everything about the report during our training, but we have a little difficulty in the field to apply it because people don't have the same assimilation skills. We help each other to better understand what we didn't understand in the training room.” *– CHW, Endline FGD, Intervention*

At endline, all CHWs in the intervention group reported transmitting data on time last month; however, less than half reported that they entered all of last month’s data in the tablet (46.8%). Specifically, among those who saw a child in the last month (n = 62), 56.5% entered data for a sick child; among those who followed up with a newborn (n = 60), 46.7% entered last month’s data for newborn follow-up; among those who provided family planning care (n = 60), 46.7% entered last month’s data for family planning; among those who led health talks (n = 62), 37.1% entered last month’s data for health talks; and among those who saw pregnant women and/or newborns (n = 60), 35% entered last month’s data for them (data not shown). Monitoring visits to intervention sites demonstrated malfunctioning tablets contributed to some CHW’s low rates of data entry.

CHWs experienced various difficulties in using the tablets related to the double workload of using both paper and digital tools to record and report community health data, the tablet quality and limited network connection. Common challenges include access and review of data on their tablet after submission, lack of phone/data credit and battery not charged (**Table 4**). At times, CHWs used their own smartphones to report data using the DHIS2 app when tablets malfunctioned. Qualitative data further indicates mixed CHW experiences in using the tablets for reporting.

“Filling out our paperwork is more complicated than filling out the tablets... if you have to do all this work, it's really not easy. It's better to go straight to the tablet.” *– CHW, Endline FGD, Intervention*

“We want them to give us a very good brand name tablet and a very good application. You'll see that the CHWs will work even better. If our tablets are out of charge, then it's hard to turn them back on. That's not normal. At 30% if you don't charge, you're in trouble.” *– CHW, Endline FGD, Intervention*

Overall, CHWs appreciated the data validation features in the tablet that prevent incomplete or contradictory data from being entered and saved. In contrast, CHWs noted that daily data entry using paper forms may lead to data falsification because of the reporting burden.

“The tablet is good, you can make a mistake on the individual form, but with the tablet, if you make a mistake it is indicated immediately, and you can see your mistake.” *– CHW, Endline FGD, Intervention*

In intervention settings, stakeholders generally approve of the integration of tablets and express positive effect of using tablets on the CHWs performance and data quality and timeliness compared to the paper system, while acknowledging challenges.

“At the very beginning, we had hard copies for the CHWs to send reports. This report can take a week to a month to arrive at the district level. With the introduction of the tablet, the reports are entered on the spot and we have up-to-the-minute information.” *– Stakeholder, Endline IDI, Intervention*

“Here in Mopti, [tablet use] has greatly improved the quality of the CHWs’ work. Many have put in a lot of effort and diligence into the work, which has also brought some stability to the sites since the CHWs collect data in real-time.[...] the CHWs have become very diligent at the site level to carry out their activities and provide good information*.” – Stakeholder, Endline IDI, Intervention*

Respondents agreed that eliminating paper forms, improving technical quality of tablets, providing airtime, and fully integrating the digital data collection and reporting platform would improve its usability and quality. The CHWs reported that their preferred method for data reporting was the tablet or smartphone (91.9%). Positive opinions CHWs shared include the ease of data entry, the permanence of the data (*“it doesn’t get lost*”), and the rapidity of data reporting.

“CHW 3: The change it can bring; even a year later you can look at the number of children you have had each month.

CHW 2: The importance of the tablet is that it shows you your mistakes and you correct them; it can bring us a lot of knowledge.” – CHW, Endline FGD, Intervention

“With the tablets, it's progress, we can send our data directly. But in the past, the reports would go through the DTC before they were sent.” *– CHW, Endline FGD, Intervention*

#### Data use for decision-making

At community level, analysis and use of health data are infrequent. CHWs and DTCs describe using community-level health data to inform topic selection for CHW-led health talks or to prompt vaccination campaigns. Although the qualitative data describes low involvement of CHWs in health program decision making, the CHWs, particularly in intervention sites, do not perceive this as negative. CHWs expressed high satisfaction (88%) with their participation in health service planning (**Table 3**).

“Thanks to DHSI2 I noticed the increase of malaria in some CHW sites and I initiated an awareness campaign with mothers to explain to them the importance of sleeping under a mosquito net and to clean their surroundings.” *– DTC, Baseline IDI, Intervention*

Most CHWs reported in both rounds that they “*are not consulted when decisions are made*” for health programming, and rather “*execute the decisions made*”. Stakeholders describe routine monthly meetings for DTCs, ASACOs, and other district-level stakeholders during which the District-level DHIS2 data are reviewed, analyzed and programming decisions made; this process “*hasn’t changed*” at their level.

“During one of our meetings in my health district, it was noted that the rate of assisted childbirth is low, and together we looked for the causes of this drop in the rate [...] at the monthly meeting of the ASACO. [...] As a solution, we proposed to raise awareness.” *– Stakeholder, Baseline IDI, Comparison*

“The data is used and analyzed during the management committee meetings. The ASACOs hold monthly meetings. At each meeting, the data is projected from DHIS2 and data is interpreted by the DTC. The management committee, which includes the prefect, the mayor, all the ASACOs and all the DTCs, [and others] presents the quarterly data for decision making.” *– Stakeholder, Endline IDI, Intervention*

Overall, the roles of the stakeholders, DTCs and CHWs and the overall process for review, analysis and use of health data for decision making appear largely unchanged from baseline to endline.

## DISCUSSION

Quantitative and qualitative findings suggest that digitization of community health data collection and reporting via CHWs is feasible and, with strategic supports in place, can positively affect timeliness and completeness of data reporting. Data use, however, seems largely unchanged following the intervention and may require additional inputs targeting district and CHW user capacity to interpret data outputs alongside contextual priorities as well as a longer implementation period. Using community health data routinely in decision-making needs to be promoted and further integrated into health systems accountability processes to be effective [12,21].

Our study showed mixed effects related to tablet use for CHW collection and reporting of health data but confirmed the feasibility of integrating community-level information digitally into the DHIS2, provided that adequate infrastructure and support are available to CHWs and their supervisors to ensure sustainable and scalable implementation [22-24]. While integration of digital data collection and reporting via tablet addressed the observed bottleneck requiring manual entry of aggregate data by DTCs each month, several challenges remained. Persisting barriers to implementing DHIS2 in Mopti include deficiencies in oversight and support for CHWs, reliability of equipment and infrastructure, and duplication of reporting efforts. We were unable to eliminate the paper-based collection and reporting system in our intervention sites due to programmatic constraints and hesitancy to adopt a completely digital system from the local MOH, which led to a large increase in workload for CHWs. This limits our understanding of how digital-based systems affect CHW job satisfaction and motivation, which emerged within our qualitative findings and elsewhere [25]. Further research is needed on the impact of tablet use for data collection and reporting in the absence of the paper-based system.

While our study aimed to explore changes in data use following the introduction of tablets, the intervention did not include strategies to improve data use. As such, data continues to be reviewed monthly by DTCs and other stakeholders at district level to decide health programming (eg, holding vaccination campaigns), while CHWs use site-specific data to focus their health talks. Future efforts should emphasize improving DTC capacity to monitor electronic data from CHWs and organizing local meetings to review data in tablets. Excluding CHWs from programming decisions indicates a normalized process of limited CHW participation consistent with previous studies in East and Southern Africa that found CHWs had limited motivation to engage “beyond their regular tasks” [26]. This has implications for how CHWs see themselves in relation to the broader community health system.

This study recommends more implementation research to assess strategies that involve CHWs in district planning meetings and strengthen district stakeholder capacity to interpret data. Additional support is needed to enable actors at all levels of the health system to improve data analytics, visualization, and data use to effectively inform decision-making [27].

While findings demonstrate the acceptability of tablets for improving HIS efficiencies, inferences drawn from overall experiences of tablet use suggest that digital innovations can motivate CHWs in their work [25,28]. Technological problems identified in our study included poor network connectivity and low-quality tablets. During routine monitoring visits, AKF provided support to CHWs by troubleshooting technological challenges and providing additional informal mentorship on tablet use. Supportive supervision for CHWs and initial and adequate follow-up training on digital data collection and reporting are critically important and observed in similar studies elsewhere [26,29]. Such challenges and additional budgetary concerns related to support and HIS interoperability are common and may constrain scaling up digital health interventions in LMICs [29,30]. Future research should consider digital options if they are able to develop strategies to adapt and integrate digital strategies into existing health systems processes and to overcome technological challenges. Such strategies may have more success in urban areas as network connectivity is a common barrier to digital mobile-phone-based interventions in LMICs [31,32].

This study ought to be interpreted alongside its limitations. During implementation, AKF discovered that a fault in the DHIS2 app led to an initial problem syncing the tablets with the DHIS2 server. Once fixed, the CHWs were then asked to re-enter the data (using paper backups) for the first three months of the pilot. In addition, the poor tablet quality meant that many CHWs had malfunctioning tablets at one point or another throughout implementation. The quantitative sample size was dependent upon the number of active CHWs in the study site. For the matched quantitative analysis, we excluded CHWs for whom we only had data at either baseline or endline (likely due to turnover in the post). All survey measures were self-reported by CHWs which may be subject to some recall bias. Results are not generalizable beyond the study districts.

## CONCLUSIONS

Overall, this study showed that while CHW tablet use for data collection and reporting is feasible and desirable, changes are needed for this to be a fully-functional system. Future efforts to integrate DHIS2 data collection into community health data collection and reporting need to carefully consider how to ensure site-level availability of sufficient network connectivity; quality, compatibility and functionality of technology; and routine supportive systems for CHWs and other community health actors on data use. In Mali, implementors must collaborate closely with the MOH to ensure accuracy of digital collection and reporting tools and design a system that can function on its own without a duplicative paper form system. Taking this intervention to scale could bring improvements timeliness and completeness of community-level health data in areas where these indicators were not as high and may positively affect CHW performance and motivation.
